# Real-Time Docking Ring Detection Based on the Geometrical Shape for an On-Orbit Spacecraft

**DOI:** 10.3390/s19235243

**Published:** 2019-11-28

**Authors:** Limin Zhang, Wang Pan, Xianghua Ma

**Affiliations:** School of Electrical and Electronic Engineering, Shanghai Institute of Technology, Shanghai 201418, China; zhanglimin@sia.cn (L.Z.); xhuam@sit.edu.cn (X.M.)

**Keywords:** docking ring detection, space missions, geometrical shape, real-time detection

## Abstract

Docking ring is a circular hatch of spacecraft that allows servicing spacecraft to dock in various space missions. The detection of the ring is greatly beneficial to automatic capture, rendezvous and docking. Based on its geometrical shape, we propose a real-time docking ring detection method for on-orbit spacecraft. Firstly, we extract arcs from the edge mask and classify them into four classes according to edge direction and convexity. By developing the arc selection strategy, we select a combination of arcs possibly belonging to the same ellipse, and then estimate its parameters via the least squares fitting technique. Candidate ellipses are validated according to the fitness of the estimation with the actual edge pixels. The experiments show that our method is superior to the state-of-the-art methods, and can be used in real time application. The method can also be extended to other applications.

## 1. Introduction

With the development of space exploration, the on-orbit service [1] of automatic orbital capture and repair mechanism for faulty spacecraft [2,3,4,5] has become an urgent need. In order to achieve the automatic on-orbit service, vision system is widely used to acquire the pose of the target spacecraft. A docking ring [6] is a circular hatch of spacecraft that allows servicing spacecraft to dock in various space missions. The accurate detection of docking ring can be greatly beneficial to the target’s visual tracking, recognition [7,8] and localization [9,10,11,12,13]. The geometrical shape of the docking ring is distinctive, since it is a regular circular part on the man-made spacecraft. Besides, a circle in the scene is projected into an ellipse in the image, the docking ring can be detected by finding the ellipse in the image. The ellipse parameters are usually used as input in the pose estimation procedure, so our goal is to get the ellipse parameters in the image. It is different from conventional object detection tasks [14] that try to locate the image coordinates of the object, and draw a bounding box around the object in the image. In the past few years, researchers have utilized edge contour methods to detect ellipses. Many approaches to generate the arcs are presented, such as linking short straight lines [15,16,17,18], splitting the edge contour [19,20,21,22], or connecting the edge pixels [23,24]. Instead of exhaustive search, arcs are grouped according to their relative position and constraints on the curvature [15,16,19,20,22,23,24], or ellipse fitting error [17,18,21]. In this work, the inner boundary of docking ring is the single target ellipse to be found. This is in contrast to ellipse detection approaches finding all ellipses in the image. In practice, we must deal with several major problems. Suffering from partial occlusion, poor illumination or complex background, the on-orbit spacecraft image may be of low quality, which causes the detection more challenging. Furthermore, docking ring detection algorithm need reach high accuracy and efficiency because it is mostly just a previous step for localization, which is based on the feature correspondence between the 3D model and the 2D image.

We introduce a detection method of docking ring based on the gradient direction of edge points. Arcs are extracted from the edge mask and classified into four quadrants with edge direction and convexity. By putting forwards arc selection strategy, we select a cluster of arcs possibly belonging to the same ellipse, and then estimate its parameters via the least squares fitting method. Candidate ellipses are validated according to the fitness between the estimations and actual edge pixels. The contributions of our work are summarized in the following:

(1) Our method is a general way to detect the docking ring from on-orbit spacecraft images. Based on the geometric properties and reflection characteristics of the target, the method adapts to various types of spacecraft in the complex and changeable space environment.
(2) We develop novel arc selection strategies according to the geometric properties of the ellipse, and achieve better performance than the state-of-art approaches.

## 2. The Proposed Method

We proposed an accurate and fast detection method for docking ring, which consists of three steps: arc extraction, ellipse parameters estimation, and validity verification. Firstly, the candidate arcs are extracted from the edge image by linking the edge points. Secondly, we designed arc selection strategies to group the arcs that possibly belong to the same ellipse, and then the least square method [25,26] was used to estimate elliptic parameters efficiently. Finally, the validity of the ellipses was verified by measuring the fitness between the arc and the candidate ellipse.

### 2.1. Arc Extraction

The docking ring has different reflection characteristics from the surrounding background, resulting image brightness changes sharply. Therefore, we extracted the edge pixels to determine the boundary of the docking ring. According to the basic geometric properties of the ellipse in mathematics, the gradient direction of the ellipse edge point changes continuously. Based on this feature, we proposed a method for extracting elliptic arcs from a spatially connected point sequence. The edge points with stable gradient direction changing in the edge image were extracted and connected into arc segments. The contour of the ellipse remained in these arc segments. This step was to identify candidate arcs in the image, whose key phases were elaborated as follows.

(1) Edge detection:

Our method was mainly based on the edge information of the image. We applied the Canny edge detector [27] to the input image to get the edge set. Here the edge point $e_{i}\;=\;\left(x_{i},y_{i},\theta_{i}\right)$ is defined by its image position $\left(x_{i},y_{i}\right)$ and gradient direction $\theta_{i}$.

**Figure 1:**
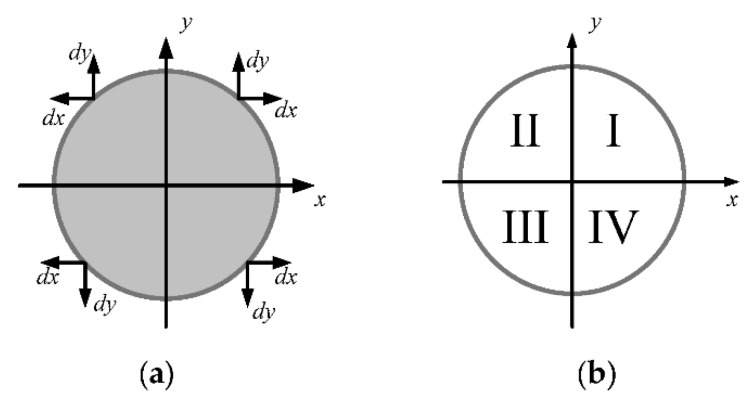
Arcs are classified into four quadrants: (**a**) the gradient direction of edge points and (**b**) arc segments.

(2) Arc segment detection:

The edge points classification is based on the grayscale difference between the target and the surrounding background. Considering the reflectance properties of the docking ring, the gray value of the region outside the contour is higher than that inside the contour, as shown in Figure 1a. According to the gradient direction of edge points, the classification of the edge point $e_{i}$ is expressed as follows:

(1) $$D\left(e_{i}\right)\;=\;\{\begin{array}{l} I,\;\;\;\;\;\;\;if\;dx>0\wedge dy>0\\ \mathrm{II},\;\;\;\;\;if\;dx<0\wedge dy>0\\ \mathrm{III},\;\;\;if\;dx<0\wedge dy<0\\ \mathrm{IV},\;\;\;if\;dx>0\wedge dy<0 \end{array}$$

where the symbol ∧ represents the logical relation “and”, and dx, dy are the derivative of Sobel in the x and y direction respectively. According to the signs of dx and dy, we divided the edge points into four categories, namely the four quadrants shown in Figure 1b.

For edge points in the same quadrant, the 8-connected edge pixels are connected into arc segments. It means that each arc $\alpha_{i}$ is a set of 8-connected edge points, and the gradient direction of its edge points is within a fixed range. Then, short arcs whose pixel number is less than the threshold $T_{p}$ were eliminated. We could acquire arc segments set {$\alpha_{i}$} of four quadrants.

**Figure 2:**
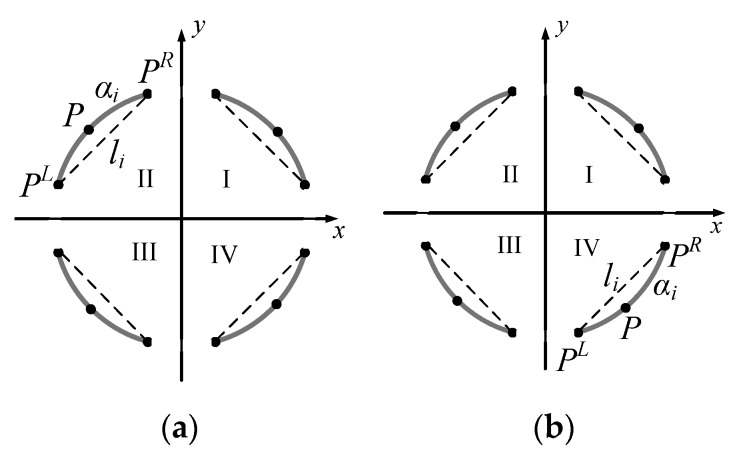
The definition of convex arc and concave arc: (**a**) the arc α*_i_* is the convex arc and (**b**) the arc α*_i_* is the concave arc.

(3) Arc segment classification:

Since the elliptic arcs located in different quadrants have different convexity, arc segments were further selected according to their convexity. The convexity was judged by the position of the point on the arc relative to the line by connecting the two endpoints, as shown in Figure 2. P represents one point on the arc segment $\alpha_{i}$, the point $P^{L}$ and $P^{R}$ are the endpoints of $\alpha_{i}$, and $l_{i}$ is the line by connecting the endpoints.

The convexity of arc $\alpha_{i}$ is represented by (2), in which “+” represents that the arc is convex, and “−” represents a concave arc. Elliptic arcs lying in the first and second quadrants are convex arcs, and the arcs lying in the third and fourth quadrants are concave arcs.

(2) $$C\left(\alpha_{i}\right)\;=\;\{\begin{array}{c} +,\;\;\left(P^{L}P^{R}\;\times \;P^{L}P\right)\cdot \left(0,0,1\right)>0\\ -,\;\;\left(P^{L}P^{R\;}\times \;P^{L}P\right)\cdot \left(0,0,1\right)<0 \end{array}$$

By considering both the edge gradient direction and the convexity, the classification of arc segments is represented in (3).

(3) $$Q\left(\alpha_{i}\right)\;=\;\{\begin{array}{l} I,\;\;\;\mathrm{if}\;D\left(e_{i}\right)=I\wedge C(\alpha_{i})=+\\ \mathrm{II},\;\;\mathrm{if}\;D\left(e_{i}\right)=\mathrm{II}\wedge C(\alpha_{i})=+\;\\ \mathrm{III},\;\mathrm{if}\;D\left(e_{i}\right)=\mathrm{III}\wedge C(\alpha_{i})=-\\ \mathrm{IV},\;\mathrm{if}\;D\left(e_{i}\right)=\mathrm{IV}\wedge C(\alpha_{i})=- \end{array}$$

This step breaks the spatially connectivity of the contour with gradient direction changing sharply, and removes some arcs that do not belong to the ellipse.

### 2.2. Ellipse Parameters Estimation

This step is to identify candidate ellipses in the image, including arc grouping and ellipse fitting.

#### 2.2.1. Arc Selection Strategy

By classifying the arc segments, the contour of a complete ellipse will be divided into four parts and located in four quadrants. We recombined the arcs from the same ellipse so as to estimate the ellipse parameters. Suffering from some challenges, including image noise, uneven illumination, and partial occlusion, arcs in real images may be fractional absence or divided into several short arc segments. In order for accurate elliptic parameter estimation, sufficient contour information is needed. Taking the above factors into consideration, arc segments of three different quadrants are selected to compose a candidate ellipse $\varepsilon_{i}$. The rules we made were robust to the possible arc fracture of the real image, and enough information was provided for the subsequent parameter estimation step. In order to effectively group three arcs that belong to the same ellipse, we developed arc selection strategies according to the geometric properties of an ellipse.

**Figure 3:**
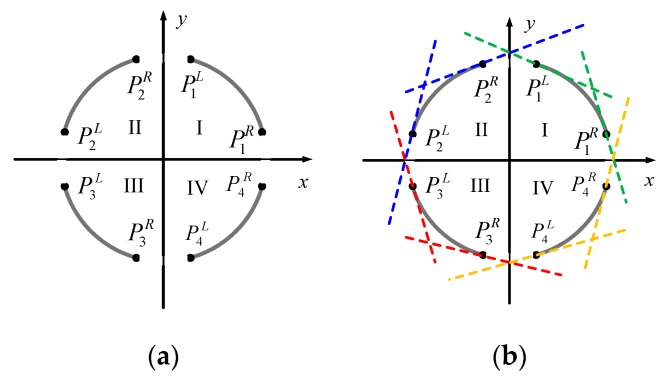
Arc selection constraints: (**a**) quadrant constraint and position constraint and (**b**) tangent constraint.

1. Arc selection for two arcs:

Firstly, the constraints for the combination of two arcs were established. Two candidate arcs $\alpha_{i}$ and $\alpha_{j}$ were considered belonging to the same ellipse if they satisfied the following three constraints:

(1) Quadrant constraint. We selected three arcs of the four quadrants to estimate an ellipse in this paper. The possible quadrants of the three arcs were indicated as follows: (I, II, III), (II, III, IV), (III, IV, I), and (I, II, IV). It shows that the three arcs were situated in three adjacent quadrants. Therefore, the combination of two arcs only combined the arcs in adjacent quadrants, as described by (4).
(2) Position constraint. Based on the quadrant constraint, if arc $\alpha_{i}$ and arc $\alpha_{j}$ belong to a same ellipse, the endpoints of the arcs are constrained by their relative position in the image, as shown in Figure 3a. Position constraint is described by (5).
(3) Tangent constraint. Assume we drew a tangent line, the ellipse was completely on one side of the tangent line. We considered the endpoint of the arc as tangent point to draw the tangent, and the tangent direction was perpendicular to the gradient direction of the endpoint, as shown in Figure 3b. Then, two arcs for combination must satisfy the constraints in (6).

(4) $$C_{q}\;=\;\{\begin{array}{l} T,\;\mathrm{if}\;\left(Q\left(\alpha_{i}\right),Q\left(\alpha_{j}\right)\right)\in \left\{\left(I,\mathrm{II}\right),\left(\mathrm{II},\mathrm{III}\right),\left(\mathrm{III},\mathrm{IV}\right),\left(\mathrm{IV},I\right)\right\}\\ F,\;\mathrm{otherwise}\; \end{array},$$

(5) $$C_{p}\;=\;\{\begin{array}{l} T,\;\;\;if\;\;\;\left(Q\left(\alpha_{i}\right),Q\left(\alpha_{j}\right)\right)\equiv \left(I,II\right)\wedge \left(P_{i}^{L}.x>P_{j}^{R}.x\right)\;\;\;\;\;\\ T,\;\;\;if\;\;\;\left(Q\left(\alpha_{i}\right),Q\left(\alpha_{j}\right)\right)\equiv \left(II,III\right)\wedge \left(P_{i}^{L}.y>P_{j}^{L}.y\right)\;\;\\ T,\;\;\;if\;\;\;\left(Q\left(\alpha_{i}\right),Q\left(\alpha_{j}\right)\right)\equiv \left(III,IV\right)\wedge \left(P_{i}^{R}.x<P_{j}^{L}.x\right)\\ T,\;\;\;if\;\;\;\left(Q\left(\alpha_{i}\right),Q\left(\alpha_{j}\right)\right)\equiv \left(IV,I\right)\wedge \left(P_{i}^{R}.y<P_{j}^{R}.y\right)\\ F,\;\;\;otherwise \end{array},$$

(6) $$C_{t}\;=\;\{\begin{array}{l} T,\;\;\;if\;\left(Q\left(\alpha_{i}\right),Q\left(\alpha_{j}\right)\right)\equiv \left(I,II\right)\wedge \left(P_{j}^{R}.y<f_{i}^{L}\left(P_{j}^{R}.x\right)\right)\wedge \left(P_{i}^{L}.y<f_{j}^{R}\left(P_{i}^{L}.x\right)\right)\;\;\;\;\\ T,\;\;\;if\;\left(Q\left(\alpha_{i}\right),Q\left(\alpha_{j}\right)\right)\equiv \left(II,III\right)\wedge \left(P_{j}^{L}.x>g_{i}^{L}\left(P_{j}^{L}.y\right)\right)\wedge \left(P_{i}^{L}.x>g_{j}^{L}\left(P_{i}^{L}.y\right)\right)\;\;\\ T,\;\;\;if\;\left(Q\left(\alpha_{i}\right),Q\left(\alpha_{j}\right)\right)\equiv \left(III,IV\right)\wedge \left(P_{j}^{L}.y>f_{i}^{R}\left(P_{j}^{L}.x\right)\right)\wedge \left(P_{i}^{R}.y>f_{j}^{L}\left(P_{i}^{R}.x\right)\right)\\ T,\;\;\;if\;\left(Q\left(\alpha_{i}\right),Q\left(\alpha_{j}\right)\right)\equiv \left(IV,I\right)\wedge \left(P_{j}^{R}.x<g_{i}^{R}\left(P_{j}^{R}.y\right)\right)\wedge \left(P_{i}^{R}.x<g_{j}^{R}\left(P_{i}^{R}.y\right)\right)\\ F,\;\;\;otherwise \end{array},$$

where $y\;=\;f_{k}^{L}\left(x\right)$ and $y\;=\;f_{k}^{R}\left(x\right)$ represent the tangent equations of the left endpoint $P_{k}^{L}$ and the right endpoint $P_{k}^{R}$ of the arc $\alpha_{k}$ respectively, the inverse equations are expressed as $x\;=\;g_{k}^{L}\left(y\right)$ and $x\;=\;g_{k}^{R}\left(y\right)$.

**Figure 4:**
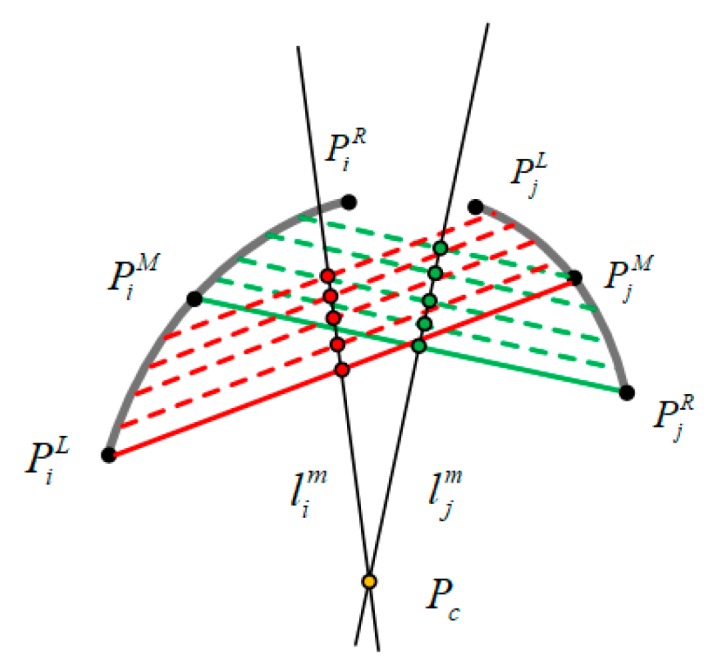
Ellipse center calculation.

For arcs $\alpha_{i}$ and $\alpha_{j}$ that satisfy the above constraints, the center of the ellipse was calculated, as shown in Figure 4. We connected the endpoint $P_{i}^{L}$ of arc $\alpha_{i}$ and the middle point $P_{j}^{M}$ of $\alpha_{j}$ into a line. Then we created multiple parallel lines, and fitted a line $l_{i}^{m}$ to the middle points of the parallel lines. Similarly, $l_{j}^{m}$ was another fitted line. The intersection $P_{c}$ of two straight lines $l_{i}^{m}$ and $l_{j}^{m}$ was the estimation of the ellipse center.

2. Arc selection for three arcs:

Next, the rule of combining three arcs was made. Three candidate arcs would be grouped, if the following constraints were satisfied:

(1) Any two of three arcs in the adjacent quadrant met the arc selection constraints for two arcs. There were two groups of arc pair in the adjacent quadrant, each group of arcs needed to meet the arc selection for two arcs.
(2) Constraints on the center of the ellipse. Three arcs originated from the same ellipse had the same ellipse center. To avoid the effect of the image noise, the centers of the ellipses calculated by two groups of arcs were required to be located within a preset distance.

#### 2.2.2. Parameter Estimation for Candidate Ellipse

When dealing with 5D ellipse parameters estimation, the voting mechanism of Hough transform is time consuming. So, we adopted the least square method to get the conic expression of the ellipse, and then acquired the 5D parameters of the ellipse through the conic expression. An ellipse is a special form of a conic:

(7) $$Ax^{2}+Bx^{2}+Cxy+Dx+Ey+F\;=\;0$$

By using the least square method, all points on the arcs could be fitted to the quadratic curve of (7), and then the coefficients (*A*, *B*, *C*, *D*, *E*, *F*) could be obtained.

The geometric parameters of the ellipse include the coordinates of the center $\left(x_{0},y_{0}\right)$, the angle of ellipse rotation θ, the semi-major axis a and the semi-minor axis b, and then the standard equation of the ellipse can be expressed as

(8) $${\left(\frac{\left(x-x_{0}\right)\cos\theta +\left(y-y_{0}\right)\sin\theta }{a}\right)}^{2}+{\left(\frac{-\left(x-x_{0}\right)\sin\theta +\left(y-y_{0}\right)\cos\theta }{b}\right)}^{2}\;=\;1$$

The parameters of the ellipse can be calculated by equating the corresponding coefficients in (7) and (8).

(9) $$\{\begin{array}{l} \;x_{0}\;=\;\frac{CE-2BD}{4AB-C^{2}}\;\;\;\;\;\;\;\;\;\;\;\;\\ \;y_{0}\;=\;\frac{CD-2AE}{4AB-C^{2}}\;\;\;\;\;\;\;\;\;\;\;\;\\ \;\theta \;=\;\frac{1}{2}arctan\frac{C}{A-B}\;\;\;\;\\ a\;=\;\sqrt{\frac{2}{\left|A\delta +B\delta -\frac{C\delta }{\sin2\theta }\right|}}\\ b\;=\;\sqrt{\frac{2}{\left|A\delta +B\delta +\frac{C\delta }{\sin2\theta }\right|}} \end{array}$$

where

$$\delta \;=\;\frac{1}{Ax_{0}^{2}+Bx_{0}y_{0}+Cy_{0}^{2}-F}$$

### 2.3. Validity Verification

The ellipse set was further verified to reduce the false detection rate. In this paper, the validity of candidate ellipse was evaluated according to the fitness between the estimated ellipse and the points on the arcs.

**Figure 5:**
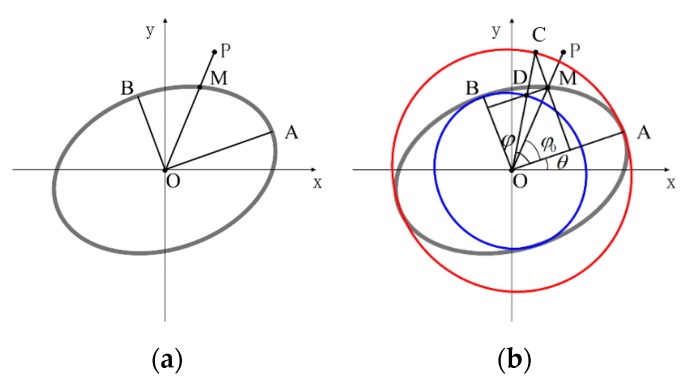
Distance from the point to the ellipse: (**a**) the definition of the distance and (**b**) the computation of the distance.

(1) Distance definition:

In this paper, the distance was calculated between pixel P on the arc and fitting ellipse E, as shown in Figure 5. We connected the edge point P and the ellipse center O, then the line OP and the ellipse E were intersected at the point M. PM is defined as the distance from the edge point to the ellipse, see Figure 5b. The distance is described in (10).

(10) $$d\left(P\right)\;=\;d_{OP}-d_{OM}$$

where,

$$d_{OM}\;=\;\sqrt{a^{2}\cos^{2}\varphi +b^{2}\sin^{2}\varphi }\;d_{OP}\;=\;\sqrt{{\left(x-x_{0}\right)}^{2}+{\left(y-y_{0}\right)}^{2}}\;\varphi \;=\;arctan\left(\frac{a}{b}\tan\varphi_{0}\right)\;\varphi_{0}\;=\;arctan\left(\frac{y-y_{0}}{x-x_{0}}\right)-\theta$$

(2) Evaluation of candidate ellipses:

The pixels on the arc of the ellipse should be located at the boundary of the estimated ellipse. Therefore, the distance between the edge point on the arc and the fitting ellipse was used to evaluate whether the candidate target meets the ellipse shape.

(11) $$d\left(P_{i}\right)\;=\;\{\begin{array}{cc} =0 & if\;P_{i}\;is\;on\;the\;boundary\;of\;E_{i}\\ <0 & if\;P_{i}\;is\;on\;the\;outside\;of\;E_{i}\;\;\;\;\\ >0 & if\;P_{i}\;is\;on\;the\;inside\;of\;E_{i}\;\;\;\;\;\;\; \end{array}$$

We could define the set $N\;=\;\left\{P_{i}:\left|d\left(P_{i}\right)\right|<T_{d}\right\}$ that contains the edge points that are close to the elliptic boundary. The score s∈[0, 1] describes how well the points of the three arcs composing $E_{i}$ fit the boundary of the estimated ellipse:

(12) $$s\;=\;\frac{N}{N_{i}+N_{j}+N_{k}}$$

where $N_{i}$, $N_{j},$ and $N_{k}$ represent the number of pixels on the three arcs respectively. The candidate ellipse $E_{i}$ satisfying $s>T_{s}$ is a valid ellipse, and the ellipse $E_{i}$ is added to the ellipse set $\left\{E_{i}\right\}$; Otherwise, $E_{i}$ is discarded which is considered as a false detection.

Finally, the uniqueness detection was carried out in the ellipse set $\left\{E_{i}\right\}$. If there were two or more ellipses with similar parameters, only the elliptic parameters with the highest score were valid.

## 3. Experiment

In order to verify the performance of the proposed algorithm, we conducted experiments both on real images and video sequences. All the experiments are executed via the C++ language on the PC equipped with an Intel(R) Core(TM) i7-7700HQ CPU@2.80 GHz processor, 8.00 GB of RAM and Windows 10 as the operating system.

### 3.1. Test on Real Images

The test on real images was to evaluate the detection effect and processing time of the proposed algorithm for the docking ring. Meanwhile, it aimed at assessing its adaptability to images of various types of spacecraft captured in the complex and changeable space environment. We compared our method with Libuda’s method [16] and Fornaciari’s method [23] on real images. The codes of the compared methods are provided by their authors. The image set contained 31 images ranging in size from 275 × 183 to 1024 × 852. The real images used in this section, including various types of spacecraft in different poses, are available on the Internet.

**Figure 6:**
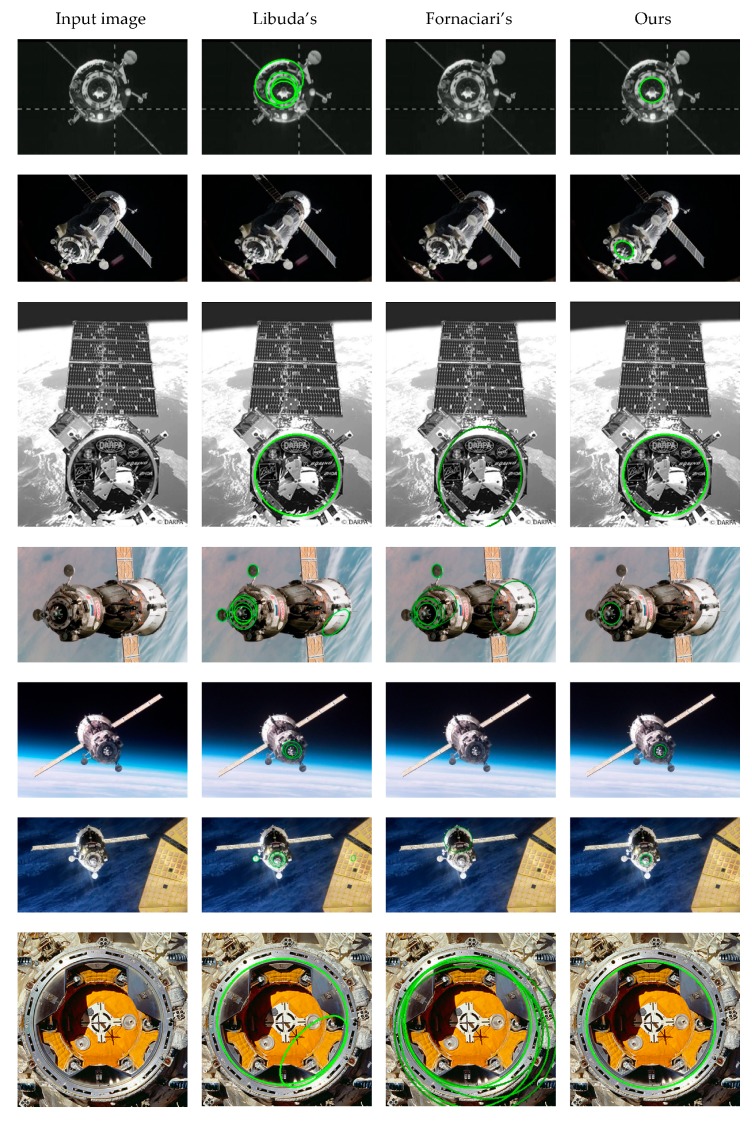

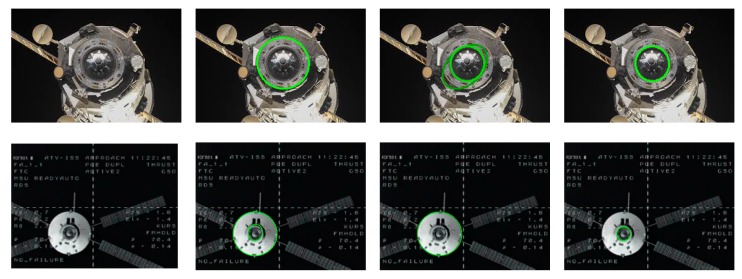
Detection results of real images.

The detection results of Libuda’s algorithm, Fornaciari’s algorithm and our algorithm are illustrated in Figure 6. The targets detected by each algorithm are marked with green ellipses. Our method can acquire a unique ellipse, which is the inner boundary of the docking ring, yet Libuda’s and Fornaciari’s algorithms have many cases of false positives and false negatives. Taking full advantage of the characteristics of the docking ring, our algorithm gets better performance than others.

**Figure 7:**
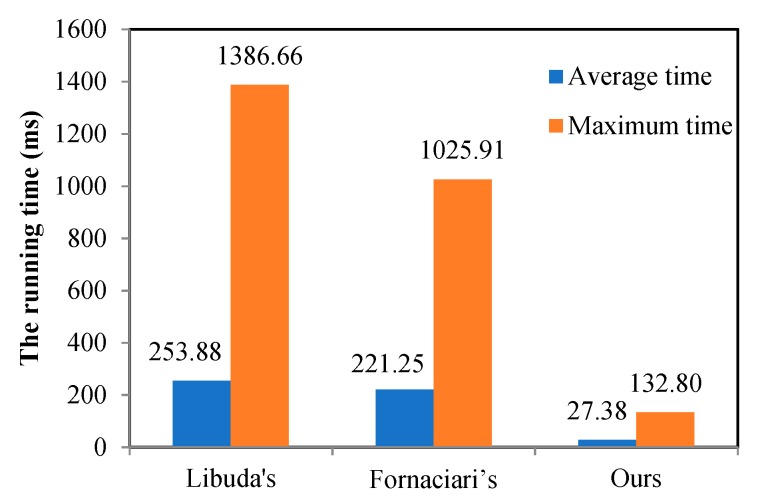
The average running time and the maximum time of the three methods.

The running time of Libuda’s algorithm, Fornaciari’s algorithm and ours in real images is shown in Figure 7. The average running time of our method was 27.38 ms and the maximum running time was 132.80 ms. Compared with the other two algorithms, our method took the least computational time, which could meet the demand of real-time application.

Libuda et al. presents a fast data driven four stage filtering process, which uses geometric features in each stage to synthesize ellipses from binary image data with the help of lines, arcs, and extended arcs. However, it suffers from a long computation time. The ellipse detector proposed by Fornaciari et al. assigns a bounding box for each arc, removes the straight edges and determines the convexity of the arc by comparing the areas of region under and over the arc. However, the method raises the detection speed at the cost of detection accuracy and robustness. The good performance of our method could be attributed to the reasonable arc selection strategy, parameter estimation, and ellipse verification. Arc selection strategy could accelerate the process of arc grouping significantly. The accuracy and efficiency of parameter estimation were guaranteed by the least square fitting technique. A reasonable ellipse validation strategy could ensure that the false detection results were filtered out. However, since our algorithm required three arcs in different quadrants in the parameter estimation of candidate ellipses, it was difficult to detect the ellipses with heavy occlusion or small ellipses that had few pixels on the boundary.

**Figure 8:**
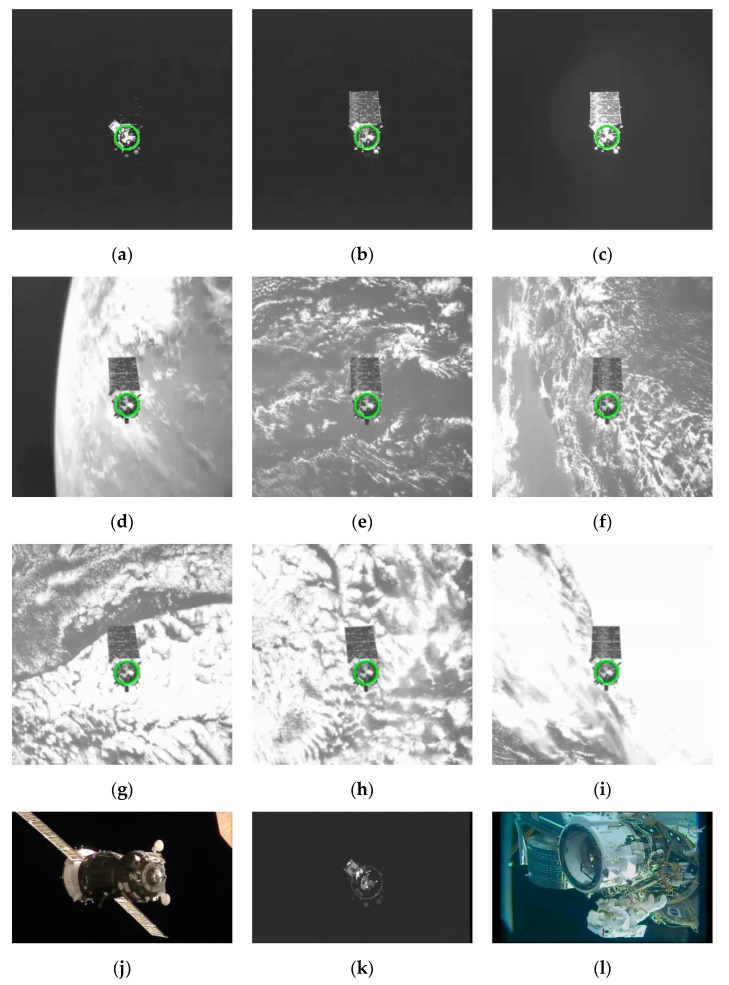
The detection results of the challenge images. (**a**–**c**) are the images under various illumination conditions; (**d**–**i**) are the images with cluttered background and image blur; (**j**–**l**) show some failures of the method.

The above experiments proved the superiority of our algorithm over other algorithms. Then we conducted experiments to verify the performance of our algorithm against illumination, complex background, and occlusion. Figure 8 shows our detection results in the challenge images. Figure 8a–c illustrates the adaptability of our method to various illumination conditions. Figure 8d–i show the robustness of our algorithm against a cluttered background and image blur. Some failures of the method are indicated in Figure 8j–l. In Figure 8k, it is difficult to extract the edge points of the ellipse because of the low contrast. In Figure 8j,l, the reason of the failure lies in the heavy occlusion and image blur.

### 3.2. Video Sequence Test

In this section, we tested the performance of the proposed algorithm on two video sequences. The video sequences were derived from the Orbital Express project and the docking mission of the Space Shuttle Atlantis to the International Space Station. The videos are available at https://www.youtube.com/watch?v=YUnQCs77PoY and https://www.youtube.com/watch?v=7M1PeXWTSSQ.

The experiments on video sequences were used to test the performance of the proposed algorithm in practical space application and the effectiveness of dealing with real docking mission. In addition, the running time of each step of the algorithm was analyzed in detail.

(1) Orbital Express project autonomously docking video sequence.

**Figure 9:**
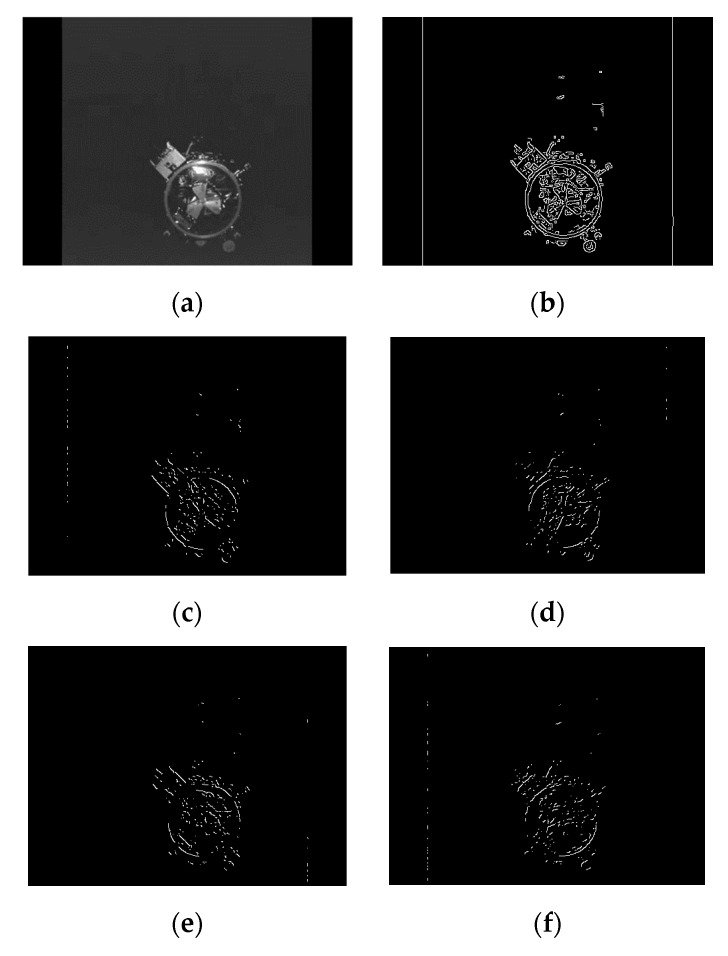

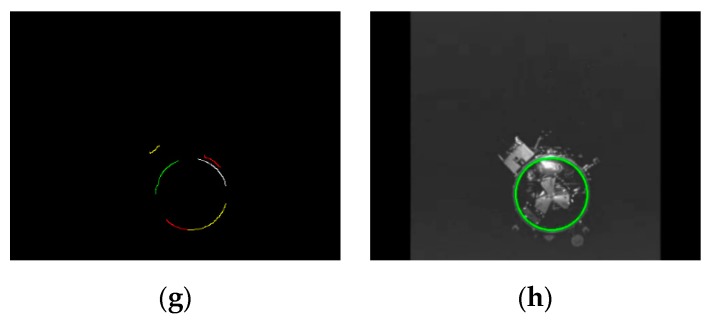
Results of the steps of our algorithm on the Orbital Express project video sequence: (**a**) the original image, (**b**) edge image, (**c**–**f**) classification of edge points by gradient direction, (**g**) the arcs after selection, and (**h**) the docking ring detection result.

The autonomously docking video sequence in the Orbital Express project [28] was used to test the effectiveness of the algorithm. The video sequence consisted of 144 images with a resolution of 480 × 360. The images were captured in the open space with no earth or other stars in the background, as shown in Figure 9a. Canny edge detection results are shown in Figure 9b. According to the gradient direction, edge points were divided into four quadrants as shown in Figure 9c–f. The similar pixel points connected in the eight adjacent areas were connected into an arc, and the arcs with a length less than the threshold are removed. The arcs shown in Figure 9g were obtained by judging the convexity. The four quadrants of arcs are represented in white, yellow, red, and green respectively. As can be seen from Figure 9g, after the steps, there were six arcs obtained for the complex image, including one arc in quadrant I, one arc in quadrant II, two arcs in quadrant III, and two arcs in quadrant IV. Finally, the detection result was obtained through ellipse fitting and validation, and the detection result in green is shown in Figure 9h.

**Figure 10:**
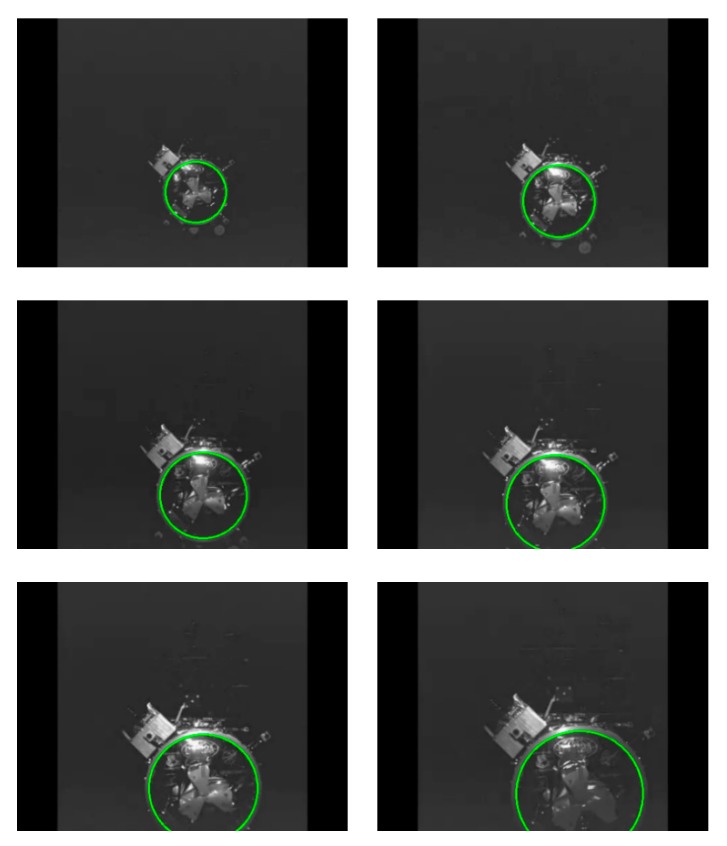
The detection results of our algorithm on the Orbital Express project video sequence.

Due to the low contrast, the edge extraction was incomplete, and the docking rings in some images were not detected. The right detection rate of our algorithm for this video sequence was 98.61%, and the average detection time was 6.96 milliseconds. Figure 10 illustrates some examples of results.

The size of each image in video sequence was consistent and the content of the image was similar, so the factors affecting the speed of the algorithm could be analyzed through the average running time of each step in the algorithm. Table 1 shows the average process time of each step of our algorithm. It could be seen that edge detection step took the most time, followed by arc detection and classification. After screening, the number of remaining arcs was small and the processing speed of subsequent steps was high.

(2) Video sequence of the Space Shuttle Atlantis docked with the International Space Station.

**Figure 11:**
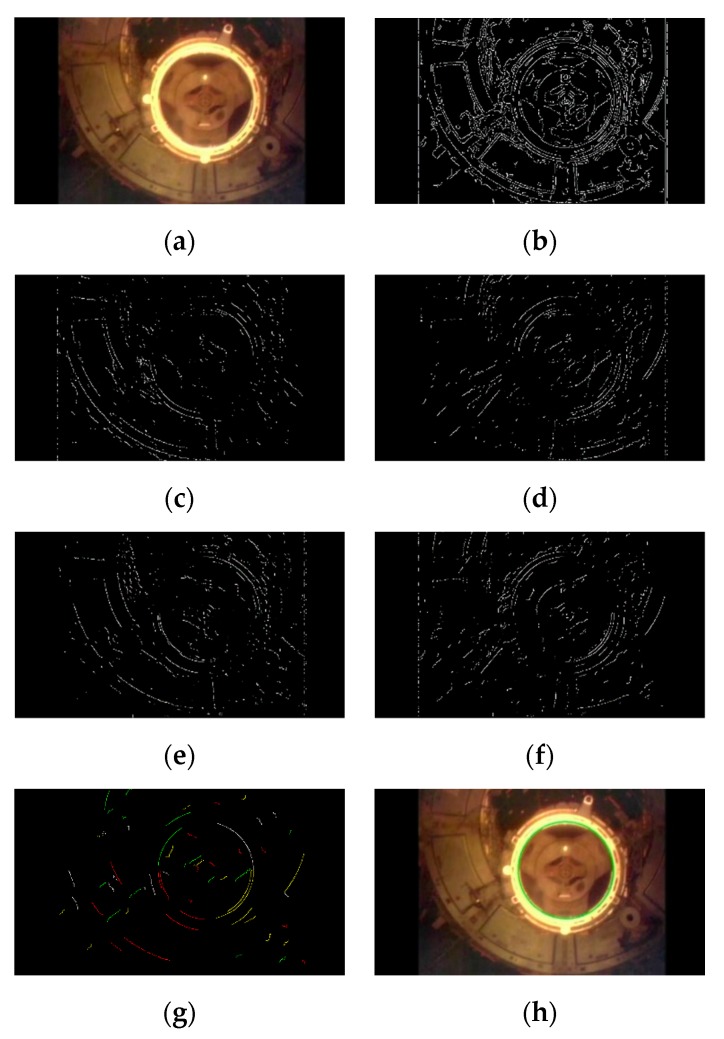
The detection results of the steps of our algorithm on Atlantis docking with the International Space Station (ISS) video sequence: (**a**) the original image, (**b**) edge image, (**c**–**f**) classification of edge points by gradient direction, (**g**) the arcs after selection, (**h**) the docking ring detection result.

The video sequence of the Space Shuttle Atlantis docked to the International Space Station (ISS) [29] was selected to further test the effectiveness of our algorithm in a space application. The video sequence included 5692 frames of images with a resolution of 852 × 480. As shown in Figure 11a, the Space Shuttle itself occupied most of the background of the image. Figure 11 shows the intermediate results, among which Figure 11b was the edge detection result, Figure 11c–f were four quadrants of edge points divided according to the direction of gradient, Figure 11g was the extraction result of four quadrants of arc segments, and Figure 11h shows the green ellipse as the final detection result.

**Figure 12:**
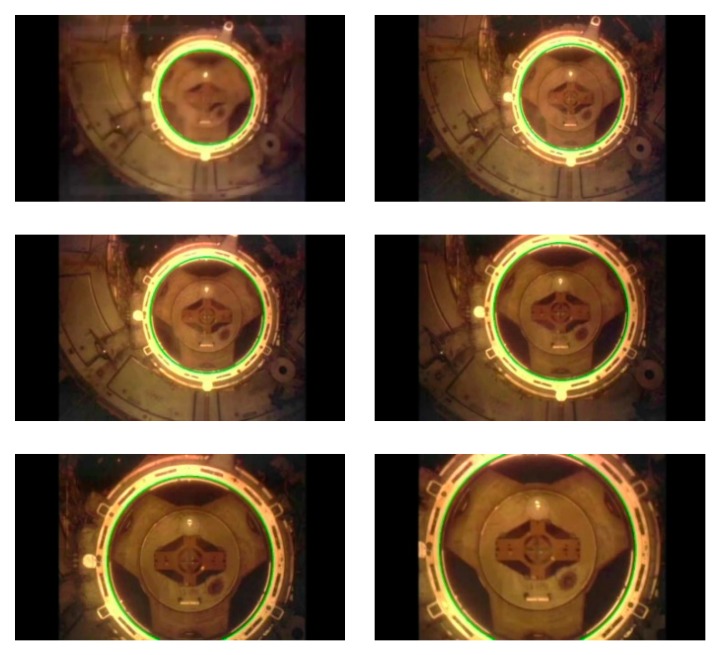
The detection results of our algorithm on Atlantis docking with the ISS video sequence.

Our algorithm achieved a 100% right detection rate for the video sequence of the Space Shuttle Atlantis docked with the International Space Station. The average detection time was 41.79 ms. Figure 12 illustrates some examples of the detection results. The green ellipse represents the detection results of our algorithm.

Table 2 shows the average processing time of each step of our algorithm. Compared with the video sequence of the Orbital Express project autonomously docking, the image resolution of this sequence was larger and the background was more complex. There were more edge points to be dealt with and the process time was longer. The running time was related to the size of the image and the complexity of image content. These two factors mainly affected the efficiency of edge detection and arc extraction. In addition, the speed of arc classification mainly depended on the threshold of arc length and the number of arcs that changing continuously in the direction of gradient. After extracting the arcs, the arc selection strategy effectively reduced the number of arc combinations and improved the accuracy, thus reducing the subsequent time of ellipse fitting and verification.

To sum up, the two video sequences verified the accuracy and efficiency of our detection algorithm, and also proved that it could be used for actual space applications.

## 4. Conclusions

In this paper, we proposed a docking ring detection algorithm based on geometrical shape from on-orbit spacecraft images. By taking advantage of the continuous change of gradient direction of the target elliptic contour, this algorithm selects pixels with relatively stable gradient direction change from the edge image and connects them into an arc to avoid the edge points with abrupt gradient direction change in the arc. On this basis, a variety of arc selection strategies are adopted to effectively reduce the number of ellipse fitting in the arc combination process, thus improving the detection efficiency. Finally, the target ellipse obtained after arc grouping is further verified to reduce the false detection rate. The method is general for spacecraft with docking rings. Experimental verification proved that our algorithm had better performance compared to other algorithms and it could be extended to other applications. Future work should investigate detecting ellipse with heavy occlusion and small ellipse.

## Figures and Tables

**Table 1:** The processing time of each step on the Orbital Express project video sequence.

Steps of the Algorithm	Time (ms)
Edge detection	4.52
Arc detection and classification	1.98
Arc grouping	0.09
Parameter estimation	0.12
Validity verification	0.24
Total time	6.96

**Table 2:** The processing time of each step on Atlantis Docking with ISS video sequence.

Steps of the Algorithm	Time (ms)
Edge detection	10.02
Arc detection and classification	8.55
Arc grouping	2.72
Parameter estimation	8.57
Validity verification	11.93
Total time	41.79

## References

[1] Flores-Abad A., Ma O., Pham K., Ulrich S. (2014). A review of space robotics technologies for on-orbit servicing. Prog. Aerosp. Sci..

[2] Huang P., Lu Y., Wang M., Meng Z., Zhang Y., Zhang F. (2019). Postcapture Attitude Takeover Control of a Partially Failed Spacecraft with Parametric Uncertainties. IEEE Trans. Autom. Sci. Eng..

[3] Li X., Tai Y., Zhang L., Li H., Li L. (2014). Characterization of dynamic random process using optical vortex metrology. Appl. Phys. B.

[4] Li X. (2012). Digital speckle correlation method based on phase vortices. Opt. Eng..

[5] Sun Q., Niu Z., Wang W., Li H., Luo L., Lin X. (2019). An Adaptive Real-Time Detection Algorithm for Dim and Small Photoelectric GSO Debris. Sensors.

[6] Finkbeiner J.R., Dunlap P.H., Steinetz B.M., Daniels C.C. (2008). Review of seal designs on the Apollo spacecraft. J. Spacecr. Rocket..

[7] Velasquez A.F., Luckett J., Napolitano M., Marani G., Evans T., Fravolini M. Experimental evaluation of a machine vision based pose estimation system for autonomous capture of satellites with interface rings. Proceedings of the AIAA Guidance, Navigation, and Control (GNC) Conference.

[8] Teutsch C., Berndt D., Trostmann E., Weber M. (2006). Real-time detection of elliptic shapes for automated object recognition and object tracking. Machine Vision Applications in Industrial Inspection XIV.

[9] Ye C., Hong S., Tamjidi A. (2015). 6-DOF Pose Estimation of a Robotic Navigation Aid by Tracking Visual and Geometric Features. IEEE Trans. Autom. Sci. Eng..

[10] Kim J.-U., Kang H.-B. (2018). A New 3D Object Pose Detection Method Using LIDAR Shape Set. Sensors.

[11] Kwon Y.C., Jang J.W., Hwang Y., Choi O. (2019). Multi-Cue-Based Circle Detection and Its Application to Robust Extrinsic Calibration of RGB-D Cameras. Sensors.

[12] Chen Z., Zhang Z., Dai F., Bu Y., Wang H. (2017). Monocular Vision-Based Underwater Object Detection. Sensors.

[13] Zhang J., Qiu Y., Duan X., Xu K., Yang C. (2019). An Improved Robust Method for Pose Estimation of Cylindrical Parts with Interference Features. Sensors.

[14] Lu K., Li J., Zhou L., Hu X., An X., He H. (2018). Generalized Haar Filter-Based Object Detection for Car Sharing Services. IEEE Trans. Autom. Sci. Eng..

[15] Kim E., Haseyama M., Kitajima H. Fast and robust ellipse extraction from complicated images. Proceedings of the IEEE Information Technology and Applications.

[16] Libuda L., Grothues I., Kraiss K.F. (2007). Ellipse detection in digital image data using geometric features. Advances in Computer Graphics and Computer Vision.

[17] Mai F., Hung Y.S., Zhong H., Sze W.F. (2008). A hierarchical approach for fast and robust ellipse extraction. Pattern Recognit..

[18] Chia A.Y.S., Rahardja S., Rajan D., Leung M.K. (2011). A split and merge based ellipse detector with self-correcting capability. IEEE Trans. Image Process..

[19] Prasad D.K., Leung M.K.H., Cho S.Y. (2012). Edge curvature and convexity based ellipse detection method. Pattern Recognit..

[20] Nguyen T.M., Ahuja S., Wu Q.M.J. A real-time ellipse detection based on edge grouping. Proceedings of the 2009 IEEE International Conference on Systems, Man and Cybernetics.

[21] Liu Z.Y., Qiao H. (2009). Multiple ellipses detection in noisy environments: A hierarchical approach. Pattern Recognit..

[22] Chen S., Xia R., Zhao J., Chen Y., Hu M. (2017). A hybrid method for ellipse detection in industrial images. Pattern Recognit..

[23] Fornaciari M., Prati A., Cucchiara R. (2014). A fast and effective ellipse detector for embedded vision applications. Pattern Recognit..

[24] Dong H., Sun G., Pang W.C., Asadi E., Prasad D.K., Chen I.M. (2018). Fast ellipse detection via gradient information for robotic manipulation of cylindrical objects. IEEE Robot. Autom. Lett..

[25] Fitzgibbon A., Pilu M., Fisher R.B. (1999). Direct least square fitting of ellipses. IEEE Trans. Pattern Anal. Mach. Intell..

[26] Chaudhuri D. (2010). A simple least squares method for fitting of ellipses and circles depends on border points of a two-tone image and their 3-D extensions. Pattern Recognit. Lett..

[27] Canny J. (1986). A computational approach to edge detection. IEEE Trans. Pattern Anal. Mach. Intell..

[28] Whelan D.A., Adler E.A., Wilson S.B., Roesler G.M. DARPA Orbital Express program: Effecting a revolution in space-based systems. Proceedings of the SPIE—The International Society for Optical Engineering.

[29] Goodman J. (2006). Louis.History of Space Shuttle Rendezvous and Proximity Operations. J. Spacecr. Rocket..

